# Bioactive Compounds in Kimchi Improve the Cognitive and Memory Functions Impaired by Amyloid Beta

**DOI:** 10.3390/nu10101554

**Published:** 2018-10-20

**Authors:** Minji Woo, Mi Jeong Kim, Yeong Ok Song

**Affiliations:** 1 Department of Food Science and Nutrition and Kimchi Research Institute, Pusan National University, Busan 46241, Korea; woo07140@pusan.ac.kr; 2 Department of Food and Nutrition, Silla University, Busan 46958, Korea; mjkim@silla.ac.kr

**Keywords:** Alzheimer’s disease, amyloid beta, behavioral test, cognitive deficits, kimchi, oxidative stress, inflammation

## Abstract

This study investigated the abilities of kimchi and its bioactive compounds to ameliorate amyloid beta (Aβ)-induced memory and cognitive impairments. Mice were given a single intracerebroventricular injection of Aβ_25-35_, followed by a daily oral administration of capsaicin (10 mg·kg-bw^–1^), 3-(4′-hydroxyl-3′,5′-dimethoxyphenyl)propionic acid (50 mg/kg bw), quercetin (50 mg/kg bw), ascorbic acid (50 mg/kg bw), or kimchi methanol extract (KME; 200 mg/kg bw) for 2 weeks (*n* = 7 per group). Carboxymethylcellulose was used as a vehicle for the normal and control groups. Behavioral task tests showed that the learning and memory abilities were significantly waned by the injected Aβ_25-35_, but these cognitive deficits were recovered by the administrated KME and kimchi bioactive compounds (*p* < 0.05). The reactive oxygen species, peroxynitrite, and thiobarbituric acid reactive substances levels were lower, and the glutathione level was higher, in the KME and bioactive compound groups than in the control group (*p* < 0.05). In the KME and bioactive compound groups, the protein expression levels of antioxidant enzymes (nuclear factor (erythroid-derived 2)-like 2-regulated superoxide dismutase-1 and glutathione peroxidase) were increased, whereas those of inflammation-related enzymes (nuclear factor-kappaB -regulated inducible nitric oxide synthase and cyclooxygenase-2) were decreased (*p* < 0.05). Thus, the antioxidative and anti-inflammatory properties of bioactive compounds-rich kimchi might help to attenuate the symptoms of Alzheimer’s disease.

## 1. Introduction

Cognitive deficits with memory losses and lack of recognition are typical signs of Alzheimer’s disease (AD), which subsequently brings about a behavioral change in afflicted individuals. It is well established that accumulation of the amyloid beta (Aβ) peptide in the brain is an immediate cause of synaptic dysfunction and neuronal cell death in AD. Nonetheless, oxidative stress and inflammation are recognized as important causative factors as well [1]. Aβ peptides play an important role in the neuroinflammatory responses mediated through reactive oxygen species (ROS) generation. In the pathogenesis of AD, the amyloid-cascade hypothesis and the oxidative-stress hypothesis are considered as one concept by many authors [1]. 

Behavioral task tests are among the tools used to evaluate cognitive deficits in the mouse model of AD [2,3], with the T-maze, novel object recognition, and Morris water maze tests being the most widely applied. Specifically, the novel object recognition test [4] and the T-maze test [5] are designed to evaluate cognitive ability through observation of the subject’s exploratory behaviors toward a new object or route, respectively. The Morris water maze task test is designed to evaluate learning and memory levels, using a swimming pool with a hidden platform [6]. 

Natural products rich in polyphenols with multiple functions, in particular antioxidative activity, might potentially hinder neurodegeneration and thereby improve memory and cognitive functions through the alleviation of Aβ-induced neurotoxicity [7]. Numerous studies have shown the preventive or curable functions of natural products and bioactive compounds against AD, such as the anti-amyloidogenic activities of resveratrol, gingerols, curcumin, and gallate in models of the disease [8]. Quercetin has also been reported for its neuroprotective effects mediated through the attenuation of oxidative stress [9] and inflammation [10] and reduction of proteins related to AD [10].

Kimchi, a Korean traditional fermented vegetable, contains numerous bioactive compounds because it is prepared with cabbage, red pepper, garlic, ginger, green onion, and fermented fish sauce. Consequently, kimchi has demonstrated various health-beneficial effects, including the prevention of oxidative stress [11], inflammation [12], and cancer [13], and improvement of cognitive abilities [3]. These effects of kimchi might be attributed to the antioxidative activity of its component bioactive compounds [14]. In our previous study, several bioactive compounds were identified from the kimchi methanol extract (KME); namely, ascorbic acid, capsaicin, 3-(4′-hydroxyl-3′,5′-dimethoxyphenyl)propionic acid (HDMPPA), and quercetin, at 280, 270, 40, and 20 mg/kg-KME, respectively [15]. Capsaicin, ascorbic acid, and HDMPPA are monophenol compounds, whereas quercetin is a polyphenol compound. In this study, the neuroprotective effects of KME and its bioactive compounds were investigated in a mouse model of Aβ_25-35_-induced AD. Behavioral task tests were performed in addition to biochemical evaluation of the protein expression of antioxidants and inflammatory cytokines in the treated animals.

## 2. Materials and Methods 

### 2.1. Kimchi Bioactive Compounds and Kimchi Methanol Extract

The individual kimchi bioactive compounds that were previously identified by our team to be present in KME [15] were purchased from Sigma-Aldrich (St. Louis, MO, USA); namely, HDMPPA (CDS011403), quercetin (Q4951, purity ≥ 95%), ascorbic acid (A5960, purity ≥ 99%), and capsaicin (M2028, purity ≥ 95%). The KME was prepared for the present study as described below.

Kimchi was first made by mixing together the following ingredients: Brined cabbage (84.9%), red pepper powder (2.6%), garlic (2.5%), green onion (2.3%), ginger (0.5%), fermented fish sauce (3.0%), sugar (0.5%), and glutinous rice paste (3.7%). The mixture was fermented at 10 °C for a day and stored at 0 °C in a kimchi refrigerator for 14 days (R-K182PM; LG, Seoul, Korea) until the pH reached 4.3 ± 0.1. The resultant kimchi was then freeze-dried (SFDSM06; Samwon Co., Busan, Korea). The KME was prepared by three repeated extractions of the freeze-dried kimchi with 70% methanol, for 24 h at room temperature each time. All extracts were combined together, concentrated using a rotary evaporator (R-200; Buchi, Flawil, Switzerland), and then freeze-dried. 

### 2.2. Animals and Intracerebroventricular Injection of Aβ_25-35_

**Figure 1:**
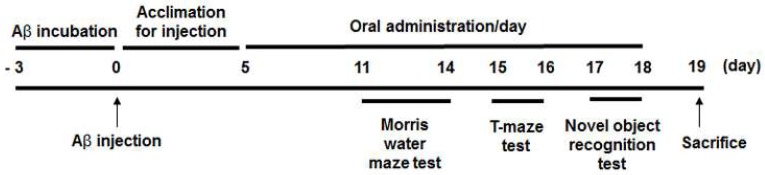
Experimental schedule for the mouse injections with Aβ_25-35._

ICR mice (male, 5 weeks old) were purchased from Orient, Inc. (Seongnam, Korea). The mice were kept under a controlled temperature (23 ± 1 °C) and humidity (50 ± 5%) with a 12 h light-dark cycle, and fed chow diet and water *ad libitum*. After 1 week of acclimatization, the animals were assigned to seven groups (*n* = 7 per group) on the basis of body weight (bw). Aβ_25-35_ (Sigma-Aldrich) dissolved in phosphate-buffered saline (PBS) was incubated at 37 °C for 3 days. On day 0, the mice in the experimental groups were given an intracerebroventricular injection of aggregated Aβ_25-35_, except for the animals in the normal (NOR) group, which were injected with PBS instead. In brief, a 5 μL Aβ_25-35_ solution (5 nmol/mouse) or PBS was injected into the bregma (2.2 mm depth) of each mouse, using a 10 μL Hamilton microsyringe equipped with a 26-gauge needle [1,16]. Then, from day 5 onward, capsaicin (at 10 mg/kg bw/day), HDMPPA, quercetin, ascorbic acid (at 50 mg/kg bw/day each), or KME (at 200 mg/kg bw/day), each dissolved in carboxymethylcellulose, was orally administered to the mice for 2 weeks. The NOR group and an Aβ_25-35_-injected control (CON) group received the carboxymethylcellulose vehicle only. The treatment dosage was determined according to previous studies [10,17,18], where the dosage of capsaicin was further adjusted since the compound demonstrated toxicity at a high dose [19]. The dosage of KME was based on that used in previous studies at which it exhibited positive effects in the mouse model [2,15]. From day 14, three different behavioral tests were performed for 8 days according to previous studies [1,2,20]; that is, 4 consecutive days for the Morris water maze test, 2 days for the novel object recognition test, and another 2 days for the T-maze test. On day 19, all mice were fasted for 12 h and then sacrificed after CO_2_ anesthetization. The experimental schedule for the animal study is shown in Figure 1. The organs were perfused with ice-cold PBS and excised. The animal protocols were reviewed and approved by the Institutional Animal Care and Use Committee of Pusan National University (Approval No. PNU-2016-1385).

### 2.3. Morris Water Maze Test

Memory function was examined with a slightly modified Morris water maze test [6]. The circular pool used for the test was 100 cm in diameter and 35 cm in height. During the experiment, the water temperature was maintained at 22 ± 1 °C. For the behavioral test, the water pool was divided into quadrants. A platform (8 cm in diameter) was placed 1 cm underneath the water at one designated quadrant, and visual signs were marked on the other quadrants for spatial navigation. White paint (water-soluble, nontoxic) was added to make the water opaque. A total of 12 trials were conducted, three times a day. The time taken to search for the hidden platform was recorded up to 60 s. After each performance, the mouse was allowed to rest on the platform for 15 s to remember the comfort of the environment regardless of mission completion. On day 4, the 10th trial was performed as usual, using a hidden platform. However, the 11th trial was carried out without the platform. The times taken for staying to find the target quadrant were measured, respectively. For the final (12th) trial, the performance test was carried out in clear water to investigate whether the latency time would be different under the more visible environment. 

### 2.4. Novel Object Recognition Test

Recognition of a new object was tested using a black-colored cage (40 × 40 × 40 cm) [4]. For the 1st trial, two identical objects (A, A’) were placed in the box, and the frequency of touching each object was recorded for 10 min, separately. In the 2nd test conducted after 24 h, one of the old objects used in the 1st trial was replaced with a new object (A, B). Novel object recognition was calculated as the ratio of the number of times touching the familiar object or new object to the sum of the touching frequencies.

### 2.5. T-maze Test

Memory function was examined using a T-maze test [5]. The T-shaped black box had two routes (the left and right arms, respectively) and a starting position at the front pouch. The left-hand-side door was designed to be open all the time, and the right-hand-side door was closed in the 1st test but opened in the 2nd test. The frequencies of touching the gate for exploring each route were recorded for 10 min. The time lap between the two trials was 24 h.

### 2.6. Reactive oxygen species, Peroxynitrite, Thiobarbituric Acid Reactive Substances, and Glutathione Levels in the Brain

Brain homogenates were prepared in PBS (1:9, *w/v*). The thiobarbituric acid reactive substances (TBARS) levels in the homogenates were determined using a malondialdehyde standard curve, and the glutathione (GSH) levels were measured using a GSH standard curve [15]. 

A post-mitochondrial fraction was obtained by centrifugation of the brain homogenate, and the ROS and peroxynitrite levels were determined in this fraction using 2′,7′-dichlorofluorescein diacetate [21] and dihydrorhodamine 123 buffer [22], respectively. Changes in the fluorescence of the reaction samples were measured at 480 and 535 nm for 30 min with a fluorescence plate reader (FLUOstar OPTIMA; BMG LABTECH, Ortenberg, Germany).

### 2.7. Western Blot Analysis

The activities of antioxidative and anti-inflammatory enzymes in the brain tissue were determined by western blot assay as described previously [23]. Enzyme protein expression was visualized by enhanced chemiluminescence, measured with a CAS-400 system (Core Bio, Seoul, Korea), and calculated using ImageJ software (National Institutes of Health, Bethesda, MD, USA). The amount of each target protein was normalized to that of alpha-tubulin according to band density. The primary antibodies to nuclear factor (erythroid-derived 2)-like 2 (Nrf2, sc-13032), superoxide dismutase-1 (SOD1, sc-11407), glutathione peroxidase (GPx, sc-133160), nuclear factor-kappaB (NF-κB, sc-109), inducible nitric oxide synthase (iNOS, sc-651), and cyclooxygenase-2 (COX-2, sc-1747) were purchased from Santa Cruz Biotechnology (Santa Cruz, CA, USA). The donkey polyclonal secondary antibody to rabbit IgG (ab6802), goat polyclonal secondary antibody to mouse IgG (ab6789), and donkey polyclonal secondary antibody to goat IgG (ab6885) were purchased from Abcam Inc. (Cambridge, UK).

### 2.8. Statistical Analysis

Values are presented as the mean ± standard deviation. Significant differences between the CON group and each experimental group were assessed by one-way analysis of variance followed by Dunnett’s multiple-comparison test, using SPSS version 22.0 software (SPSS Inc., Chicago, IL, USA). Student’s *t*-test was also applied to the data from the object recognition task test and T-maze task test. A value of *p* < 0.05 was considered statistically significant. 

## 3. Results

### 3.1. Morris Water Maze Test

**Figure 2:**
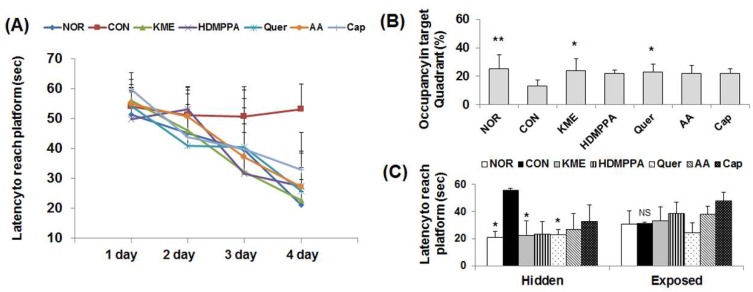
Effects of kimchi methanol extract and kimchi bioactive compounds on mouse behavior in the Morris water maze test. (**A**) The latency time required to find the platform for 4 days. (**B**) Occupancy in the target quadrant without the platform on the final test day. (**C**) The latency time required to reach the hidden and exposed platforms on the final test day. Data are the mean ± SD (*n* = 7 each group). * *p* < 0.05, ** *p* < 0.01 versus the CON group. ^NS^ No significance. (NOR: normal; CON: control; KME: kimchi methanol extract; HDMPPA: 3-(4′-hydroxyl-3′,5′-dimethoxyphenyl)propionic acid; Quer: quercetin; AA: ascorbic acid; Cap: capsaicin)

Body weight changes among the Aβ_25-35_-injected groups were not significantly different. The time taken by the experimental groups to reach the hidden platform decreased considerably with increased training time, compared with that taken by the CON group (Figure 2A). The latency time to the platform was the longest for the CON group, followed by the capsaicin, ascorbic acid, HDMPPA, quercetin, KME, and NOR groups, in order. The occupancy time in the target quadrant where the platform was originally placed was longer for the experimental groups than for the CON group, but significant difference was found only with the quercetin and KME groups (*p* < 0.05, Figure 2B). However, there were no significant differences in the latency to reach the exposed platform among the experimental groups indicating that the improvement of memory function is not associated with the visual ability (Figure 2C). 

### 3.2. Novel Object Recognition Task

**Figure 3:**
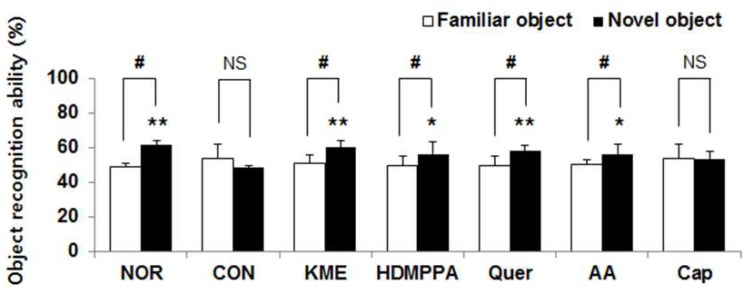
Effects of kimchi methanol extract and kimchi bioactive compounds on mouse behavior in the object recognition test. Data are the mean ± SD (*n* = 7 each group). * *p* < 0.05, ** *p* < 0.01 versus the CON group. ^NS^ No significance. ^#^ The cognitive abilities for recognizing original and novel objects were significantly different as determined by Student’s *t*-test (*p* < 0.05).

The Aβ_25-35_-injected CON group exhibited a poor ability to recognize the new object (Figure 3). Compared with that observed in the CON group, cognitive function with respect to the frequency of touching the novel object was significantly increased in the KME group, followed by the quercetin, HDMPPA, and ascorbic acid groups, in order (*p* < 0.05). The effect of KME, quercetin, HDMPPA, and ascorbic acid groups were higher by 119.5% (*p* < 0.01), 116.9% (*p* < 0.01), 114.2% (*p* < 0.05), and 114.2% (*p* < 0.05), compared with that of CON group. The CON and capsaicin groups showed no differences in recognition of the different objects. 

### 3.3. T-Maze Test

**Figure 4:**
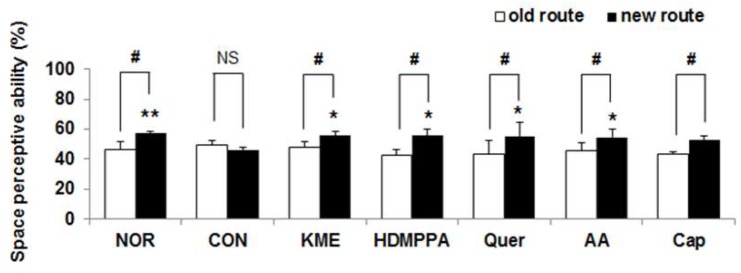
Effects of kimchi methanol extract and kimchi bioactive compounds on mouse behavior in the T-maze test. Data are the mean ± SD (*n* = 7 each group). * *p* < 0.05, ** *p* < 0.01 versus the CON group. ^NS^ No significance. ^#^ The spatial perception abilities for old and new routes were significantly different as determined by Student’s *t*-test (*p* < 0.05).

The KME group demonstrated the highest frequencies of entering a new gate to find the maze, followed by the HDMPPA, quercetin, ascorbic acid, and capsaicin groups. In particular, the KME and quercetin groups showed significantly greater spatial cognition than did the CON group (*p* < 0.05, Figure 4). 

### 3.4. Inhibition of Oxidative Stress in the Brain Tissue

The concentrations of ROS, peroxynitrite, and TBARS in the brain tissue of the CON mice were significantly higher than those of the NOR mice, whereas the GSH level was lower (*p* < 0.05, Table 1). Compared with their levels in the CON group, the ROS and peroxynitrite levels in the quercetin group were 42.7% and 52.3% lower, respectively (*p* < 0.05). The TBARS level of the KME group was the lowest, followed by that of the quercetin and HDMPPA groups, of which the concentration was decreased by 34.6%, 23.0%, and 22.8%, respectively, relative to the CON group level. In contrast, the GSH level was significantly increased in the KME and quercetin groups, by 104.2% and 103.7%, respectively (*p* < 0.05), relative to the level in the CON group.

### 3.5. Elevation of the Antioxidative Status in the Brain Tissue

**Figure 5:**
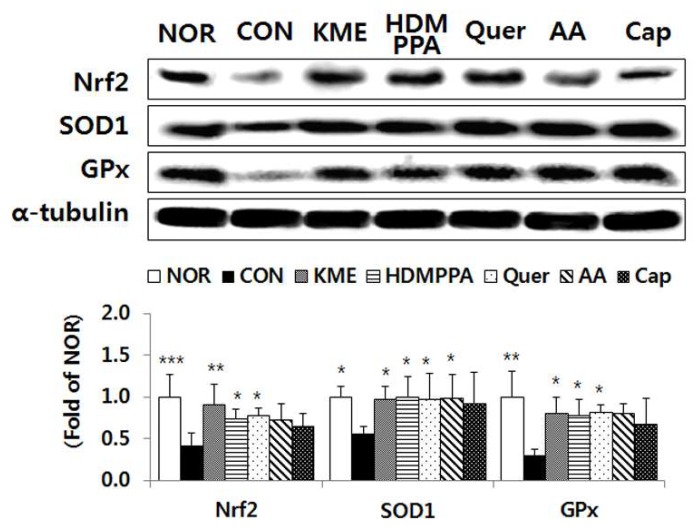
Protein expression levels of antioxidant enzymes in brain tissue of Aβ_25-35_-injected ICR mice. Data are the mean ± SD (*n* = 7 each group). * *p* < 0.05, ** *p* < 0.01, and *** *p* < 0.005 versus the CON group.

Compared with its level in the CON group, the protein expression level of the transcription factor Nrf2 was significantly elevated in the KME, HDMPPA, and quercetin groups, by 213.4% (*p* < 0.01), 172.8% (*p* < 0.05), and 181.7% (*p* < 0.05), respectively (Figure 5). The protein expression level of SOD1 was also significantly higher in the KME, HDMPPA, quercetin, and ascorbic acid groups, by 174.4%, 179.7%, 175.1%, and 176.8%, respectively (*p* < 0.05). In addition, the protein expression level of GPx was significantly higher in the KME, HDMPPA, and quercetin groups, by 272.3%, 262.5%, and 274.9%, respectively (*p* < 0.05).

### 3.6. Suppression of the Inflammatory Response in the Brain Tissue

**Figure 6:**
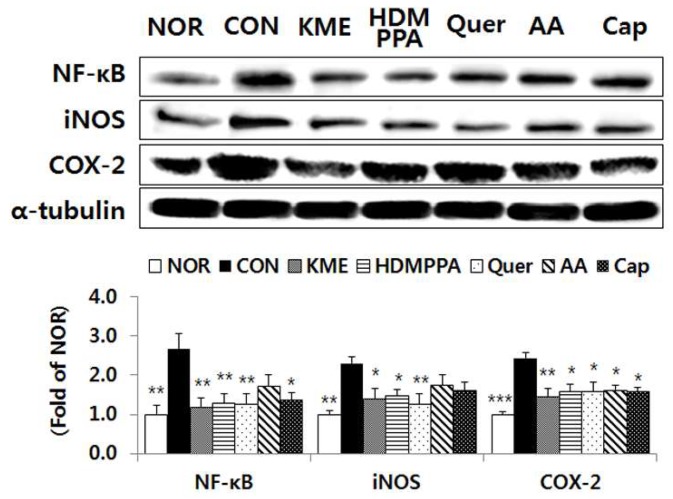
Protein expression levels of inflammation-related molecules in brain tissue of Aβ_25-35_-injected ICR mice. Data are the mean ± SD (*n* = 7 each group). * *p* < 0.05, ** *p* < 0.01, and *** *p* < 0.005 versus the CON group.

Compared with its level in the CON group, the protein expression level of the transcription factor NF-κB was significantly reduced in the KME, HDMPPA, quercetin, and capsaicin groups, by 55.7% (*p* < 0.01), 51.6% (*p* < 0.01), 52.4% (*p* < 0.01), and 48.6% (*p* < 0.05), respectively (Figure 6). The expression level of iNOS was also significantly lower in the KME, HDMPPA, and quercetin groups, by 39.1% (*p* < 0.05), 36.1% (*p* < 0.05), and 45.3% (*p* < 0.01), respectively. In addition, the protein expression level of COX-2 was significantly lower in the KME, HDMPPA, quercetin, ascorbic acid, and capsaicin groups, by 40.7% (*p* < 0.05), 35.3% (*p* < 0.01), 35.2% (*p* < 0.01), 34.2% (*p* < 0.01), and 35.6% (*p* < 0.01), respectively.

## 4. Discussion

AD is the most representative of the age-related progressive neurodegenerative disorders that lead to cognitive deficits [1,2]. Behavioral changes are commonly noted in individuals suffering from AD because cognitive function impairment accompanies a loss of memory or lack of recognition. In the present study, mouse behavior that reflects cognitive function was significantly waned by the injected Aβ_25-35_ peptide. However, the reduced learning and memory abilities were recovered by the administered KME and kimchi bioactive compounds, where the latency time to find a hidden platform was decreased, and the abilities to recognize the novel object and new route were promoted. The beneficial effects of KME and kimchi bioactive compounds against AD might be attributed to their antioxidative and anti-inflammatory activities, which inhibit senile plaque formation as well as oxidative stress in the AD-afflicted brain. In the present study, administration of the KME and kimchi bioactive compounds to Aβ_25-35_-injected mice inhibited oxidative stress in the brain. ROS and peroxynitrite generation and TBARS production were reduced with a concomitant increase of the GSH level and Nrf2-regulated antioxidant enzymes. Moreover, we had previously observed that treatment of Aβ_25-35_-injected mice with KME or kimchi bioactive compounds reduced the levels of amyloid precursor protein (APP), beta-secretase (BACE), and tau proteins in the brain [24]. Blood–brain barrier (BBB) penetration of the natural products is a primary requirement for their ability to protect against degenerative neural diseases. Our team previously examined the BBB penetration ability of some kimchi bioactive compounds and KME, where the in vitro permeability (*P*e) values of capsaicin, quercetin, HDMPPA, and KME were 68.3, 14.4, 4.7, and 0.6 × 10^−6^ cm/s, respectively, indicating that these bioactive compounds and KME could penetrate the BBB successfully [24]. The permeability of the BBB is controlled by its physicochemical characteristics, and thus hydrophobic and small molecules can readily diffuse into the brain regions [25]. 

Another critical factor for AD pathology is neuroinflammation injury, the occurrence of which is strongly associated with oxidative stress in the brain. In patients with AD, the Aβ peptides induce elevation of the inflammatory cytokines in the brain [26], suggesting that non-steroidal drugs with anti-inflammatory effects could reduce AD risk. In the present study, the KME and kimchi bioactive compounds suppressed the protein expression levels of inflammatory cytokines regulated by NF-кB. In particular, those effects were significantly higher in the KME, HDMPPA, and quercetin groups. Bioactive compounds with antioxidative activity, including phenolic compounds like curcumin and quercetin, have also been shown to mediate neuroprotective effects through the alleviation of oxidative stress [27]. Quercetin diminished the inflammatory response by suppressing NF-κB-mediated cytokine generation in vitro [27,28], in human astrocytes [29], and in the brain of d-galactose-treated mice [30]. Similarly, HDMPPA reduced the protein expression level of inflammatory enzymes such as COX-2 and iNOS in mice [31].

The neuroprotective effects of quercetin, HDMPPA, and KME were higher than those of the other bioactive compounds, possibly as a result of their structure specificity. Bioactive compounds that have demonstrated neuronal cell protection against oxidative stress are hydrophobic and have a typical structure, such as a hydroxyl group on the C3 position of the unsaturated C ring [32,33,34]. Among the bioactive compounds used in this study, quercetin contains a hydroxyl group on the C3 position, and HDMPPA and capsaicin also have a hydroxyl group on the phenol ring. Moreover, quercetin, HDMPPA, and capsaicin are hydrophobic compounds. In addition, all three are phenol-containing compounds, and the antioxidative activity of compounds with phenol moiety is well established [34]. However, the behavioral task test results from our current study do not correlate with the in vitro *P*e value. Despite capsaicin having the highest *P*e value [24] among the tested compounds, it showed no neuroprotective effects in the AD mice in this study. The possible explanation could be the low dosage (10 mg/kg bw/day) of capsaicin administered to the mice to avoid lethal toxicity. Because the oral LD_50_ value of capsaicin is 118 mg/kg bwafter a single dose [19], less than 10 mg/kg bw/daywas suggested for long-period study [17]. Such a low dose is likely unable to produce neuroprotective effects. In contrast, vitamin C (ascorbic acid) with the lowest *P*e value of 0.1 × 10^−6^ cm/s among tested compounds demonstrated neuroprotective effects in this study, albeit not as much as those shown by the other bioactive compounds. A previous study reported that the oxidized form of vitamin C (dehydroascorbic acid) could enter the brain and remain in brain tissue in the form of ascorbic acid [33]. Surprisingly, in our present study, the neuroprotective effects of KME in mice were as great as those of quercetin, which showed the greatest effects among the tested kimchi bioactive compounds. This might be due to the high concentration of KME (200 mg/kg bw/day) provided, on top of the synergic effects from the quercetin, HDMPPA, ascorbic acid, capsaicin, and other possible bioactive compounds in kimchi. 

Kimchi has revealed many health benefits [12,13,14] because its antioxidative potential is high, given that phenolic compounds, carotenoids, flavonoids, vitamin C, and lactic acid bacteria are present in this Korean fermented vegetable [14]. In our previous study, bioactive compounds such as quercetin, capsaicin, ascorbic acid, and HDMPPA were identified in KME [15]. Quercetin, a polyphenolic compound that is present in cabbages or onions, inhibits oxidation by scavenging free radicals [9], suppressing ROS production, and elevating the levels of SOD, GPx, and GSH [32,33]. HDMPPA, an active compound isolated from KME, exhibits antioxidative and anti-inflammatory effects by increasing the GSH concentration and decreasing ROS levels [35]. Capsaicin in red pepper is known to have antioxidative and anticancer properties [36]. In addition, vitamin C reduces ROS and nitrite production in tissues or plasma [33]. Both kimchi [2] and quercetin [20] were demonstrated to attenuate memory deficits and cognitive impairment in animal models of AD induced by Aβ_25-35_, which are in line with our results. Moreover, several studies have shown that diets rich in antioxidants demonstrate neuroprotective effects against AD [9,37,38]. Curcumin [39], ginkgo biloba [40], and quercetin [9,10,20], all with antioxidative and anti-inflammatory activities, were shown to improve spatial learning and memory abilities by reducing the level of the AD biomarkers APP, BACE, or Tau protein. 

In summary, we found that KME and several of its bioactive compounds improved the cognitive functions of mice with Aβ_25-35_-induced AD, possibly due to their antioxidative and anti-inflammatory activities. Our results could support the agreement among researchers that the amyloid-cascade and the oxidative-stress hypotheses of AD can be considered as one concept [1]. Further studies on the mechanisms of action of the antioxidative and anti-inflammatory compounds capsaicin and ascorbic acid and their association with neuronal diseases are needed. We conclude, however, that the daily consumption of kimchi might be a healthy practice to prevent the onset of AD. 

## Figures and Tables

**Table 1:** Reactive oxygen species, peroxynitrite, thiobarbituric acid reactive substances, and glutathione levels in the brain of Aβ_25−35_-injected ICR mice.

Group	ROS	Peroxynitrite	TBARS	GSH
	(Flu/min/mg Tissue)		(mM/g Tissue)	
NOR	999 ± 82 **	582 ± 91 *	78 ± 27 ***	24.8 ± 0.5 **
CON	2143 ± 336	1338 ± 103	138 ± 20	23.2 ± 0.7
KME	1258 ± 192 *	654 ± 193 *	90 ± 16 ***	24.6 ± 0.2 *
HDMPPA	1287 ± 321 *	693 ± 228 *	107 ± 7 *	23.9 ± 0.4
Quer	1228 ± 335 *	637 ± 346 *	107 ± 8 *	24.8 ± 0.8 *
AA	1539 ± 466	648 ± 157 *	116 ± 14	24.1 ± 1.2
Cap	1650 ± 604	774 ± 465	120 ± 4	23.3 ± 0.5

Data are the mean ± SD (*n* = 7 each group). * *p* < 0.05, ** *p* < 0.01, and *** *p* < 0.005 versus the CON group. (NOR: normal; CON: control; KME: kimchi methanol extract; HDMPPA: 3-(4′-hydroxyl-3′,5′-dimethoxyphenyl) propionic acid; Quer: quercetin; AA: ascorbic acid; Cap: capsaicin; ROS: reactive oxygen species; TBARS: thiobarbituric acid reactive substances; GSH: glutathione)
